# Determination of the water gap and the germination ecology of *Adenanthera pavonina* (Fabaceae, Mimosoideae); the adaptive role of physical dormancy in mimetic seeds

**DOI:** 10.1093/aobpla/ply048

**Published:** 2018-08-23

**Authors:** Ganesh K Jaganathan, Kirsty J Yule, Matthew Biddick

**Affiliations:** 1 Department of Biothermal Engineering, University of Shanghai for Science and Technology, Shanghai, China; 2 School of Biological Sciences, Victoria University of Wellington, Wellington, New Zealand

**Keywords:** *Adenanthera pavonina* (Fabaceae: Mimosoideae), artificial burial, summer temperatures, water gap

## Abstract

Dormancy caused by impermeable seed coats, i.e. physical dormancy (PY), regulates the timing of seed germination in species of several genera belonging to 18 angiosperm families. Physical dormancy also occurs in some mimetic species whose seeds mimic brightly coloured, fleshy fruits or arilled seeds. However, the conditions that break dormancy, as well as the location of water gaps in mimetic seeds, remain unclear. Here, we investigated the adaptive role of impermeable coats in the mimetic seeds of *Adenanthera pavonina* (Fabaceae: Mimosoideae). Specifically, we explored: (i) the conditions that break PY; (ii) the location of the primary water gap that forms during dormancy break; and (iii) the effect of seasonal temperature regimes on seed germination. Seeds were subjected to hot-water treatment, rapid temperature fluctuations and storage at temperatures mimicking summer and autumn conditions. Seed coat anatomy and water-gap regions were characterized using scanning electron microscopy (SEM) and light microscopy. Seeds were artificially buried in the field at 3 and 7 cm depths and exhumed every 6 months for 2 years to monitor germination. *Adenanthera pavonina* had impermeable seed coats, and thus PY. Seeds treated with hot water and exposed to summer–autumn temperature regimes broke dormancy. Water entered only through the lens (Type-II simple) due to dislodgement of the palisade layer. Seeds buried at 3 cm depth had significantly higher germination than those buried at 7 cm depth, with germination primarily occurring in autumn. Seeds required high summer temperatures followed by moderate autumn temperatures to become permeable to water and germinate in the field during the wet season. We conclude that the impermeable seed coat of *A. pavonina* is an adaptation that synchronizes germination with the growing season.

## Teaser

Several hypotheses have been described for the evolution of impermeable seed coat, i.e. physical dormancy (PY) in mimetic-seeded species. However, no attempts have been made to understand the importance of dormancy in controlling germination timing in mimetic species. Thus, in this study, we investigated this knowledge gap using *Adenanthera pavonina* and found impermeable seed coats are indeed a form of dormancy and evolved to control germination timing in mimetic species, although future studies on other species are required.

## Introduction

Dormancy caused by impermeable seed coats, i.e. physical dormancy (PY), regulates the timing of seed germination in several genera of 18 angiosperm families globally (Baskin and Baskin 2014). Impermeable seed coats develop during maturation drying when the palisade layer of lignified Malphian cells become impermeable to water and, occasionally, gases (Baskin *et al.* 2000; Jaganathan 2016). Dormancy delays germination under otherwise favourable conditions (e.g. high moisture, light and temperature) that would stimulate germination if seeds were non-dormant (Egley and Chandler 1983; Morrison *et al.* 1998). Physical dormancy is broken when specific structures in the seed coat, i.e. ‘water gaps’, open, allowing imbibition of the embryo (Baskin and Baskin 2014). Environmental changes, such as seasonal temperature fluctuations, fire, or passing through the gut of an animal can break dormancy, causing germination to occur in the subsequent growing season when conditions are favourable (Baskin and Baskin 2014; Jaganathan 2015; Jaganathan *et al.* 2016). Physical dormancy also occurs in some mimetic species whose seeds mimic brightly coloured, fleshy fruits or arilled seeds (Peres and van Roosmalen 1996). Despite much research, the adaptive role of impermeable seed coats in mimetic species remains largely unknown.

Mimetic seeds occur in multiple plant families but are disproportionately common in Fabaceae (Ridley 1930; McKey 1975). Multiple evolutionary functions have been proposed to explain the role of impermeable coats in mimetic species. Ridley (1930) suggested that hard, impermeable seed coats protect the embryo from damage when granivorous birds use them as grit for grinding other seeds within their gizzard. However, Galetti (2002) disputed the ‘grit hypothesis’, showing that mimetic seeds consumed by birds, with or without muscular gizzards, had lower germination rates than seeds that were not ingested. Galetti (2002) instead hypothesized that the impermeable seed coats of mimetic seeds mimic more nutritionally rich species and thereby increase dispersal rates (i.e. deceptive dispersal). Although some support for the false consumption of mimetic seeds exists (Burns 2005), it is primarily young, naïve birds that engage in consumption, and consequently dispersal, while adults tend to ignore seeds due to their lack of nutritional reward (Barrows *et al.* 1980; Andrieu and Debussche 2007). More recently, Brancalion *et al.* (2010) hypothesized that the impermeable seed coat of mimetic species evolved to prevent seed deterioration during extended attachment to the mother plant, effectively maintaining seed viability whilst increasing dispersal time. However, consensus about the role of the impermeable seed coat in mimetic seeds is yet to be reached. Furthermore, surprisingly little is known about PY in mimetic seeds, or the conditions that break it.

The anatomical changes that produce water gaps in mimetic seeds during dormancy break are currently unknown. However, the anatomical structures that open as water gaps in non-mimetic Fabaceae vary between three subfamilies and within genera (Dell 1980; Morrison *et al.* 1998; Delgado *et al.* 2014; Rodrigues-Junior *et al.* 2014a; Jaganathan *et al.* 2017). Whilst the lens becomes the water gap in several species of Papilionoideae (Hagon and Ballard 1970; Mott and McKeon 1979; Manning and Van Staden 1987; Morrison *et al.* 1998; Karaki *et al.* 2012), Caesalpinioideae (Lersten *et al.* 1992; Geneve 2009; de Paula *et al.* 2012; Rodrigues-Junior *et al.* 2014b) and Mimosoideae (Hanna 1984; Serrato-Valenti *et al.* 1995), other structures including the hilar slit can also serve as a primary water entry point (Hu *et al.* 2008; Delgado *et al.* 2014). Heating seeds of *Albizia lophantha* (Mimosoideae) resulted in the eruption of the strophiolar plug adjacent to the hilum, which acts as a water gap (Dell 1980). Likewise, the strophiole plug was the primary water gap for *Acacia kempeana* seeds when exposed to heat (Hanna 1984).

Water gap formation is irreversible and directly influences the timing of seed germination. The germination ecology of non-mimetic seeds with PY has received considerable attention. For instance, burial experiments with PY seeds in the tropics showed that high summer temperatures play an important role in breaking dormancy, with seeds germinating during the following wet season (Probert 2000; Jaganathan and Liu 2014). However, our understanding of the germination ecology of mimetic seeds is comparatively poor. Peres and van Roosmalen (1996) compared the germination percentage of untreated and scarified mimetic seeds of *Ormosia coccinea* buried in the soil for 1 year under natural field conditions finding that, concurrent with similar experiments on non-mimetic seeds, untreated mimetic seeds did not germinate during the study, yet 45 % of scarified seeds germinated within 4 months of being in the soil. Likewise, scarified mimetic seeds of *Ormosia lignivalvis* germinated to a higher percent compared with untreated seeds (Peres and van Roosmalen 1996). In south-eastern Peru, scarified seeds of mimetic *Ormosia macrocalyx* and *Ormosia bapiensis* germinated during the erratic rainfall of the dry-to-wet season transition, causing seedlings to die from subsequent drought (Foster 2008). However, seeds of *O*. *macrocalyx* and *O. bapiensis* with intact seed coats germinated only during the rainy season with most seedlings surviving, which indicates the impermeable seed coat of mimetic seeds may effectively control germination timing (Foster 2008).

*Adenanthera pavonina* is a 6–15 m tall deciduous tree native to southern China and India (Fleischmann 1997). Commonly used in agroforestry for nitrogen fixation and animal fodder, *A. pavonina* was widely introduced and naturalized in south-east Asia, Africa, and many Pacific and Caribbean islands (Fleischmann 1997). Its seed pods are ventrally dehiscent, containing 8–12 hard coated seeds, vivid red in colour and impermeable to water (Brancalion *et al.* 2010). Seeds are 7.5–9 mm in diameter and adhere to the pod, often remaining on the parent plant for prolonged periods (Brancalion *et al.* 2010). In this study, we investigated the role that the impermeable seed coat plays in the germination ecology of *A. pavonina*. Specifically, we explored: (i) the conditions that break PY in *A. pavonina*; (ii) the anatomical changes that occur in the seed coats when dormancy is broken, with particular emphasis on structures that form the primary water gap; and (iii) the germination ecology of *A. pavonina* seeds in a long-term burial experiment in the field, assessing the relationship between germination and seasonal environmental regimes.

## Methods

### Study site and seed collection

Mature seeds of *A. pavonina* were collected during May–July 2013 from Tamil Nadu in the Western Ghats of India (11°10′N, 76°74′E). The region has a dry, tropical climate with a mean annual temperature of 26.6 °C, ranging from 23.9 °C in December to 29.7 °C in May. The study site receives an annual rainfall of 646.8 mm, mostly falling during October and November. *Adenanthera pavonina* seeds are dispersed by wind between January and May and fully mature seeds detach from the pods in the soil. Seeds normally germinate after autumn when the rainfall increases. Several hundred seeds were collected from 23 randomly chosen *A. pavonina* trees located within a 15-km radius during May–June 2013. Because seed coat impermeability is only induced during the final stages of maturation drying, seeds remaining attached to trees could be permeable to water. Thus, only seeds that were dislodged when trees where gently shaken were considered mature and impermeable. Seeds were cleaned in the laboratory, pooled and stored in glass jars in ambient conditions [23–26 °C, 50–60 % relative humidity (RH)] until used in experiments. A proportion of seeds were air-freighted to the University of Shanghai for Science and Technology, Shanghai, China for laboratory investigations. The time between collection and receipt of seeds in China was 13 days and experiments began immediately after receipt. The remaining seeds were used in field experiments at the collection site within 1 week of collection.

### Seed mass and moisture content

Seed mass was determined immediately after collection by measuring five replicates of 100 seeds using a digital balance. The moisture content of fresh seeds was determined by drying four replicates of 25 seeds at 103 ± 2 °C for a period of 17 ± 1 h and reweighing (ISTA 2015). The moisture content of intact seeds was calculated as the difference between fresh and dry weight.

### Imbibition

To investigate *A. pavonina*’s seed coat impermeability, the percentage increase of water imbibed over 96 h was measured using three replicates of 25 seeds for untreated seeds, manually scarified seeds (using a razor blade) or seed dipped in boiling water (100 ± 2 °C for 45 s). After weighing, seeds were placed on moistened filter paper in 90 mm Petri dishes. Seeds were removed from the Petri dishes at 8-h intervals, blotted to remove excess water, reweighed and returned to Petri dishes. Water was added as required to keep the filter paper moist. An increase in seed weight therefore indicates an increase in water imbibed by seeds.

### Seed germination

The germination ability of untreated and seeds treated with hot water was tested under four temperature regimes. Three replicates of 25 seeds each were sown on 1 % distilled water agar and incubated for 30 days at alternating temperatures of 15/20, 20/25, 20/30, or 25/30 °C and at 15/25 °C (complete darkness; achieved by wrapping the Petri dishes with aluminium foil). Each group was exposed to 12 h light supplied by white fluorescent tungsten lamps, with an irradiance of ~30 µmol m^−2^ s^−1^ between 400 and 700 nm, synchronized with the warm temperature phase. All seeds were examined daily for germination and were considered germinated when the radicle had emerged 2 mm or more (ISTA 2010). Seeds in the dark treatment were examined for germination in a dark room and were not exposed to light during the germination period. Seeds still not germinated after 30 days were subjected to a cut test.

### Empirical dormancy breaking treatments

The breaking of dormancy opens water gaps that facilitate imbibition (i.e. water absorption by the embryo). To investigate conditions that break dormancy, freshly collected, intact *A. pavonina* seeds were subjected to one of the following treatments:

(1) *Hot-water treatment*: Three replicates of 25 seeds per treatment were dipped in water heated to either 40, 50, 60, 70, 80, 90 or 100 °C for 45 s and allowed to cool to room temperature. The seeds were then placed on moist filter paper in 90 mm Petri dishes and monitored for germination under ambient room conditions (~25 °C).
(2) *Room temperature storage*: Four replicates of 50 seeds were stored in Petri dishes at room temperature (22 °C) for 2 years, then tested for imbibition. Seeds that remained impermeable when tested at the end of storage were dipped in boiling water for 45 s and investigated for germination to assess seed viability.
(3) *Relative humidity*: Four replicates of 50 seeds were either stored in a dry room (15 % RH and 15 °C) or above water in an air-tight container (~100 % RH at room temperature) for 1 year, then tested for imbibition. At the end of storage, seeds were dipped in boiling water for 45 s and tested for germination to assess seed viability.
(4) *Rapid temperature fluctuations*: Summer soil temperatures within the collection site fluctuate between 15 and 60 °C (Jaganathan and Liu 2014). Six replicates of 25 seeds were incubated at temperatures fluctuating between 15 and 65 °C for 60 cycles with each temperature lasting 1 h. Thus, seeds were exposed to similar temperatures prevalent in the soil, but unlike in the natural environment the duration of exposure was short. Three replicates of 25 seeds were tested for imbibition after both 30 and 60 cycles. Seeds still not germinated were dipped in boiling water for 45 s and tested for germination to assess seed viability, using the above methods.

### Role of summer and autumn temperatures in *Adenanthera pavonia* seed germination

To test whether changes in temperatures from summer to autumn break PY in mimetic seeds and lead to germination, intact seeds were stored in summer conditions, i.e. in dry Petri dishes at 15/60 °C (10/14 h photoperiod) for either 1 or 2 months. Three replicates of 25 seeds were then moved to autumn conditions of either 15/45 °C (10/14 h photoperiod) or 30 °C constant temperature (10/14 h photoperiod) for 1 month. All the seeds were then assessed for germination in ambient conditions. Seeds that remained ungerminated at the end of treatments were dipped in boiling water for 45 s and tested for germination to assess seed viability.

### Seed coat anatomy and water gap identification

Seed coat anatomy was examined in intact seeds. Seeds were cut longitudinally across the hilum and examined using an infinity metallographic light microscope (Model LW300-LJT, Cewei, China). To determine the changes in seed coats during dormancy break, untreated seeds and hot-water-treated seeds were coated with gold in a Technics Hummer VI sputter coater. The hilum side of all seeds was scanned in an FEI Quanta 450 field emission scanning electron microscope and examined for changes in seed coats.

### Water entry route

To investigate the primary route of water entry into mimetic seeds, the hilum, micropyle or lens areas were blocked with white petroleum jelly and the differences in seed mass after imbibition testing were examined. Intact seeds were dipped in boiling water for 45 s to break dormancy. Four replicates of 20 seeds had either the hilum–micropyle, lens–micropyle or hilum–lens areas blocked with petroleum jelly, leaving only one water entry point per group of seeds. Seeds were stored on moistened filter paper in 9 mm Petri dishes and seed imbibition was determined via the difference in seed mass at 8-h intervals over a 96-h period.

### Artificial burial

To investigate natural conditions that lead to germination, dormant seeds were buried at varying depths at a field site and monitored for 2 years. Before burial, intact seeds were thoroughly mixed and subjected to an imbibition test. Seeds that did not absorb water were considered physically dormant (impermeable seed coat). Fifty dormant seeds were placed into each of 24 nylon mesh bags (*n* = 1200) and three bags placed into each of eight plastic trays (24 cm × 12 cm × 3 cm). Trays had water drainage holes and were filled with soil from the field site after debris was removed. All trays were buried at a random location within the collection site in August 2013. Trays were buried to a depth of 3 cm (*n* = 4) and 7 cm (*n* = 4) with temperatures recorded at hourly intervals during the burial period (August 2013 to August 2015) using a calibrated data logger (Tinytag plus 2; Gemini Data Loggers Ltd, UK).

One tray containing three nylon mesh bags was removed from each depth in February 2014, August 2014, February 2015 and August 2015. The contents were spread on a laboratory bench and visually inspected for germinated seeds. Seeds not germinated from each bag created a new replicate (i.e. 50 minus the number of seeds germinated in soil) and were incubated at 20/25 °C under light conditions in 1 % agar-water for 4 weeks. Seeds were assessed for germination daily during the 4-week period. Any seeds still not germinated after 4 weeks were dipped in hot water for 45 s and incubated under the aforementioned germination conditions.

### Statistical analysis

To test for a difference between the varying temperatures of hot-water treatments and the percentage of seeds that broke dormancy, we performed a one-way ANOVA with *post hoc* Tukey test. The germination percentage at different temperatures was analysed using a one-way ANOVA with *post hoc* Tukey test. The difference in percentage of seeds that germinated during artificial burial experiments in the field was analysed using a GLM ANOVA with burial depth and duration of burial as the fixed factors, and germination percentage as the dependent factor. Data were *arcsine* transformed to improve normality when assumptions of ANOVA were not met. All analyses were conducted in SPSS software (v. 21.0).

## Results

### Seed mass and moisture content

The average mass of 100 fresh, intact *A. pavonina* seeds was 30.6 ± 3 g (mean ± SD). Average seed moisture content was 7.8 ± 1.2 %.

### Imbibition

**Figure 1.**
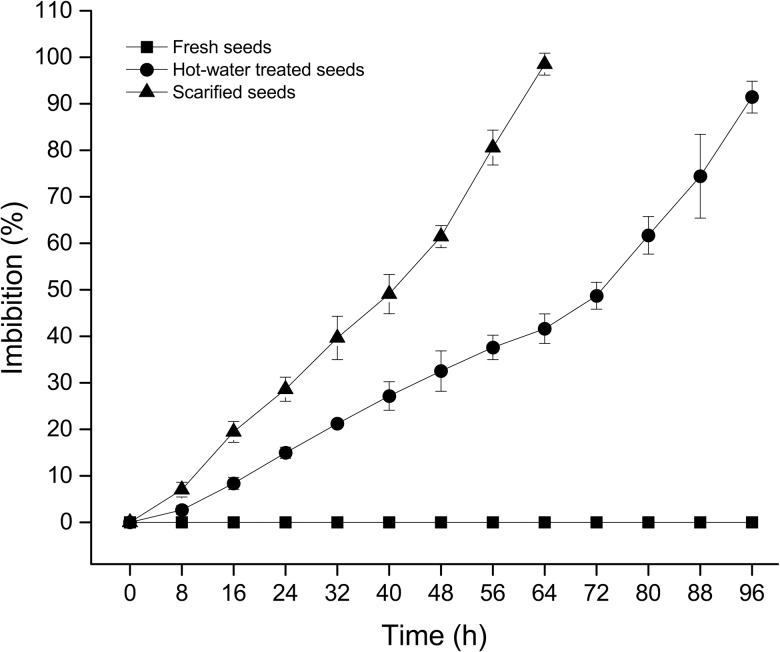
Mean percentage increase in seed mass is plotted against time for untreated, mechanically scarified and hot-water-treated mimetic seeds of *Adenanthera pavonina* sown on moist filter paper and stored in ambient laboratory conditions. Error bars represent the standard deviation of the mean.

Fresh, intact seeds were tested for an impermeable seed coat via their imbibition ability and compared to seeds treated with mechanical scarification or hot water. No untreated seeds imbibed water (Fig. 1). Both mechanical scarification and hot-water treatment resulted in imbibition and an increase in seed mass (Fig. 1). Mechanically scarified seeds imbibed water faster than hot-water-treated seeds (Fig. 1).

### Germination of fresh seeds

Freshly collected seeds did not germinate at any of the temperatures tested. However, more than 80 % of seeds treated with hot water germinated at all temperatures tested (15/20 °C = 88 ± 1.7 %, 20/25 °C = 85 ± 1.15 %, 25/30 °C = 90 ± 1.52 % or 25/30 °C = 84 ± 1.73 %). When incubated at a variable temperature of 20/25 °C under complete darkness, 79 ± 3.9 % of the seeds germinated. However, there was no statistically significant difference between germination percentage at the different temperatures tested (*P* > 0.05). A cut test on seeds that were not germinated revealed that seeds were either empty, damaged or dormancy was simply not broken, although the latter accounted for less than 5 % of seeds.

### Empirical dormancy breaking treatments

**Figure 2.**
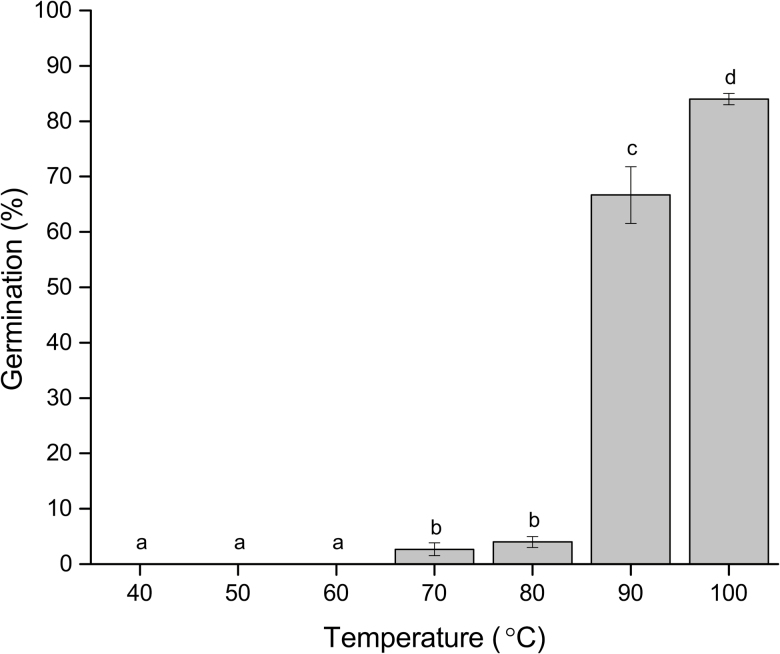
Mean percentage of *Adenanthera pavonina* seeds that broke dormancy when treated with hot water of different temperatures for 45 s. Different letters denote significant differences between treatments.

(1) *Hot-water treatment*: The temperature at which seeds were treated with hot water had a significant effect on breaking dormancy (Fig. 2, *P* < 0.05). A Tukey *post hoc* analysis showed that, although nearly 65 % of seeds dipped in 90 ± 2 °C water germinated, seeds dipped in 100 ± 2 °C water had significantly higher germination (Fig. 2, *P* < 0.05).
(2) *Room temperature storage*: Seeds stored at room temperature had 1 ± 0.5 % and 4.5 ± 2.5 % germination after 1 and 2 years, respectively. When non-germinated seeds were dipped in boiling water, 88 ± 3.5 % and 91 ± 3.7 % of seeds germinated, respectively.
(3) *Relative humidity*: None of the seeds stored under room conditions for 1 year germinated or swelled when tested for imbibition at the end of storage. However, 91 ± 2.1 % of seeds germinated after dipping in boiling water for 45 s, indicating seeds were viable yet remained dormant after treatment.
(4) *Rapid temperature fluctuations*: No seeds subjected to rapid temperature fluctuations germinated after 30 cycles. However, 1.5 ± 1 % of the seeds germinated after 60 cycles. When the remaining seeds were dipped in boiling water, 80 ± 1 % and 85 ± 2.1 % of seeds germinated after 30 and 60 cycles, respectively.

### Role of summer and autumn temperatures in *Adenanthera pavonia* seed germination

**Figure 3.**
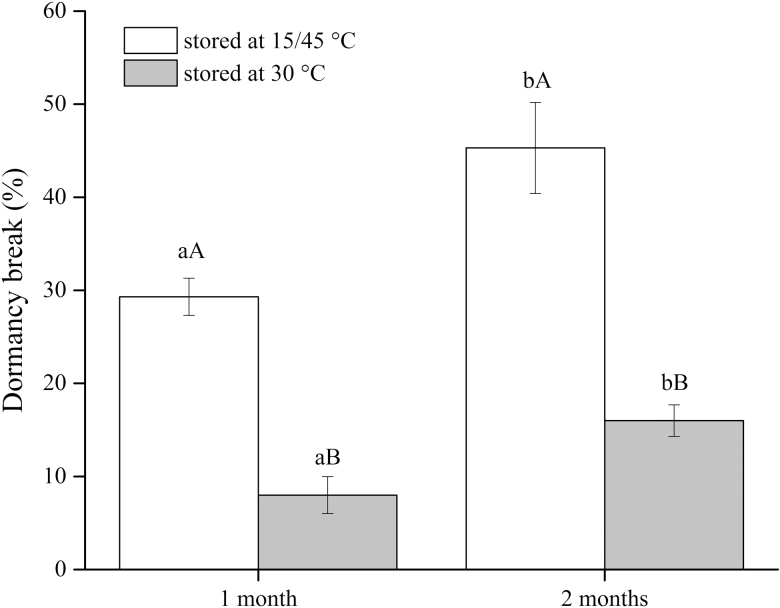
Mean percentage of *Adenanthera pavonina* seeds that broke dormancy and germinated after being stored for either 1 or 2 months at summer temperatures of 15/60 °C and subsequently moved to autumn temperatures of either 15/45 °C or constant 30 °C. Different lower-case letters denote a significant difference between storage temperature after storing at 15/45 °C. Different upper-case letters denote a significant difference between 15/45 and 30 °C after 1- and 2-month storage period.

Only 3 and 14 % of seeds stored at 15/60 °C (10/14 h photoperiod) for 1 or 2 months germinated, respectively. When the seeds were moved to autumn conditions, many seeds took up water and germinated (Fig. 3). However, at a constant temperature of 30 °C, fewer seeds imbibed water (Fig. 3). Two-month storage at summer temperature followed by 1-month storage at autumn temperature broke dormancy in 45 % of the seeds. Subsequent germination of the seeds that did not germinate during treatments reached over 80 % when dipped in boiling water for 45 s.

### Seed coat anatomy and water gap identification

**Figure 4.**
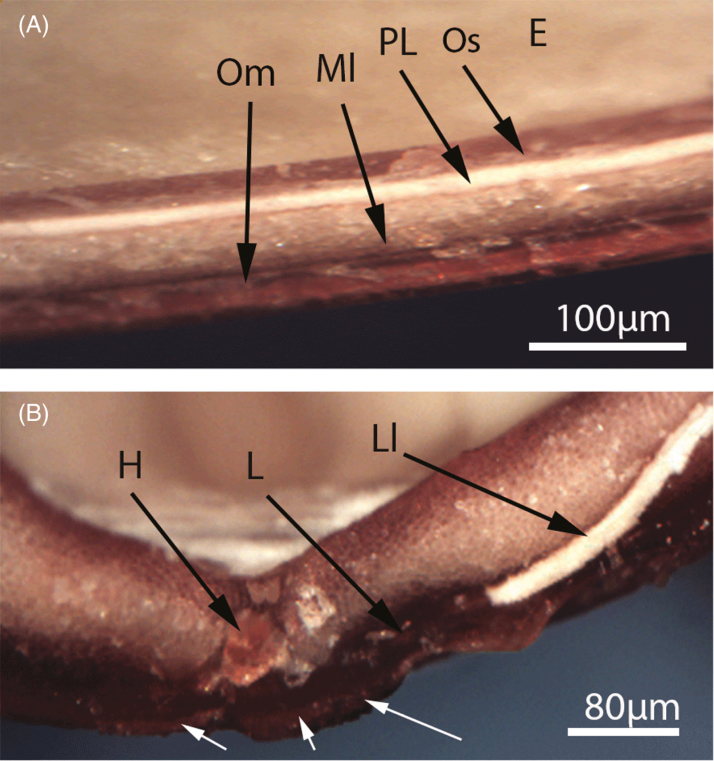
Photomicrographs of longitudinal sections of *Adenanthera pavonina* seed coat in (A) non-hilar region and (B) hilar region. E, endosperm; H, hilum; LI, light line; Ml, mesophyll layer; Om, outer macrosclerids; Os, osteosclerids; PL, palisade layer. White arrows mark the thin palisade layer on the hilar surface.

**Figure 5.**
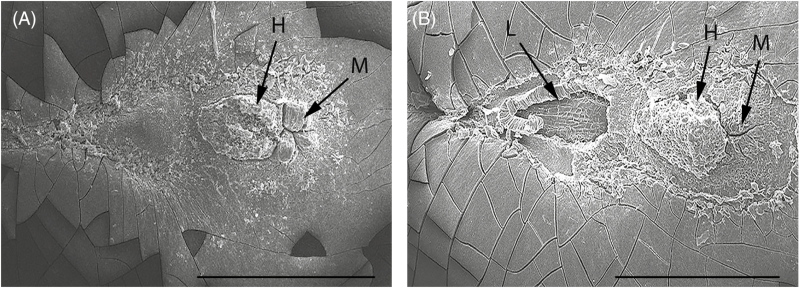
Scanning electron micrographs of (A) dormant and (B) non-dormant seeds showing the hilar side of the seeds. H, hilum; L, lens; M, micropyle. Scale: 700 µm.

Seeds contained various cell layers above the endosperm (Fig. 4A). The thickness and arrangement of cell layers differed between the hilar region and the rest of the seed coat. The palisade layer in the non-hilar region of the seed coat lies below a thick mesophyll layer and above a thin layer of osteosclerids cells. In the hilar region, the palisade layer was very thin and there was no visible light line (Fig. 4B). However, the palisade layer does occur near the seed coat surface (white arrows, Fig. 4B). This layer is blended with other cells to form the hilum. The palisade layer under the lens also appears weak and the whole structure of the lens under a scanning electron microscope appeared different compared with other parts of the seed coat (Fig. 5A).

The hilar section of the untreated seeds only has a micropyle and hilum (Fig. 5A). Hot-water treatment resulted in dislodgement of the lens, which is present on the opposite side of the micropyle (Fig. 5B). The palisade layer present under the lens region opened in a circular manner. In the control seeds, the lens area appeared different to those of other seed parts (Fig. 5A). The hilum did not undergo any changes after hot-water treatment and no visible cracks were observed (Fig. 5B).

### Water entry route

**Figure 6.**
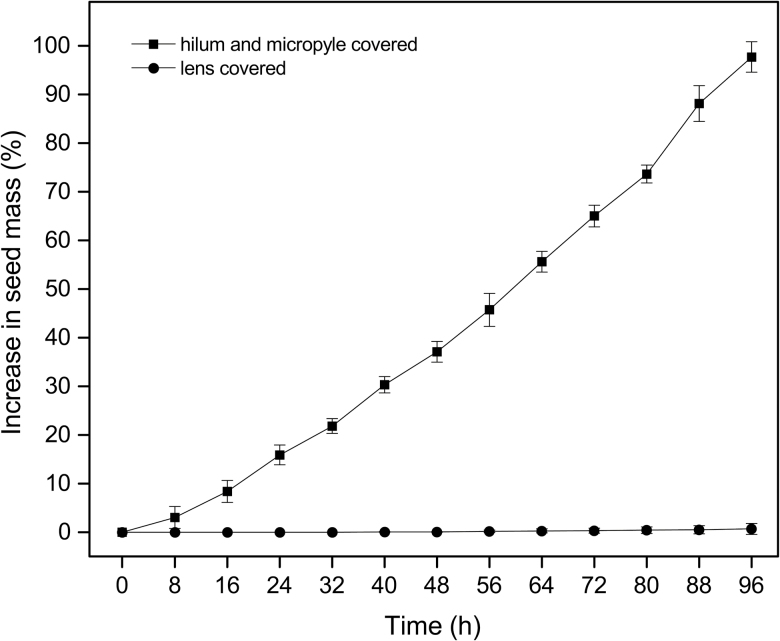
Percentage increase in mass of *Adenanthera pavonina* seeds dipped in hot water for 45 s and covered with petroleum jelly at either the lens or micropyle and hilum. Error bars represent the standard deviation of the mean.

Water imbibition of dormancy broken seeds in which the hilum and micropyle were covered reached nearly 100 % in 96 h (Fig. 6). In contrast, when the lens was covered seeds did not absorb water, even after 1 week. Only one lens-covered seed showed some imbibition out of 75 seeds tested. However, seeds began imbibing water after removing the petroleum jelly from the lens.

### Artificial burial

**Figure 7.**
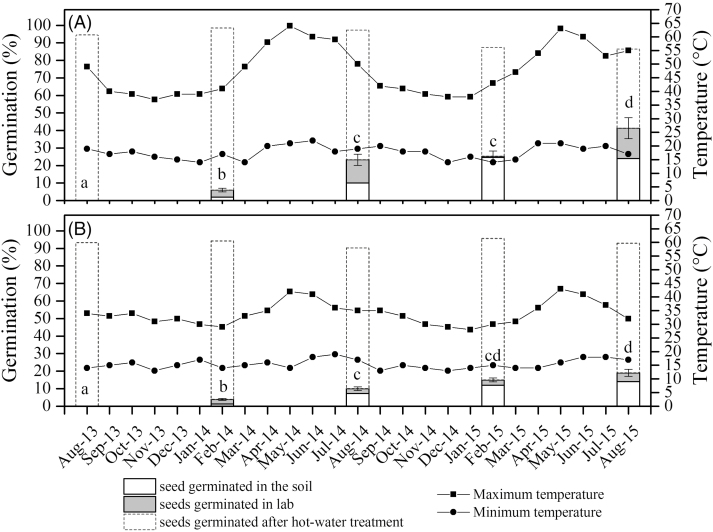
Maximum and minimum soil temperature in °C recorded at 3 and 7 cm below the soil surface at the burial site between August 2013 and August 2015. Seeds of *Adenanthera pavonina* buried at (A) 3 cm depth and (B) 7 cm depth were exhumed every 6 months from soil and assessed for germination at 20/25 °C. Germination of seeds buried in soil, germinated in the laboratory and germinated after dipping in boiling water are shown in percentages. Different lower-case letters indicate a significant difference in percentage of seeds germinated in the field and laboratory (combined).

No seeds were permeable to water prior to burial. Seeds buried at both 3 cm and 7 cm depths broke dormancy and germinated in the soil or later in the laboratory. The number of seeds that germinated after burial at 3 cm depth was significantly higher than the number of seeds that germinated after burial at 7 cm depth (Fig. 7; Table 1). This difference was observed throughout the burial period. Seeds retrieved following summer conditions exhibited higher germination rates at both depths. However, a similar percentage of seeds exhumed in early spring germinated as did seeds retrieved in summer (Fig. 7; Table 1). Soil temperature declined with depth. At 3 cm depth, the temperature rose to around 65 °C during the summer months of April to June, but remained close to 40 °C during November to January (Fig. 7). On average, there were 113 days in 2014 and 122 days in 2015 in which temperatures rose above 55 °C during summer. In contrast, the temperature never exceeded 50 °C at 7 cm depth, with minimal summer fluctuation (Fig. 7). There were 59 days in 2014 and 43 days in 2015 in which temperatures exceeded 40 °C.

**Table 1.** Results of a GLM ANOVA showing the effects of seed burial depth and burial duration on germination percentage of *Adenanthera pavonina* seeds in the soil. ^a^*R*^2^ = 0.974 (adjusted *R*^2^ = 0.962).

Source	Type-III sum of squares	df	Mean square	*F*	Significance
Corrected model	6.172^a^	9	0.686	83.545	0.000
Intercept	15.154	1	15.154	1846.135	0.000
Burial depth	0.256	1	0.256	31.162	0.000
Burial duration	5.803	4	1.451	176.748	0.000
Burial depth * burial duration	0.113	4	0.028	3.437	0.027
Error	0.164	20	0.008		
Total	21.490	30			
Corrected total	6.336	29			

## Discussion

In the present study, we investigated dormancy breaking requirements, seed coat anatomy and germination ecology in the mimetic seeds of *A. pavonina* using long-term field and laboratory experiments. We report four key findings: (i) mature seeds have an impermeable seed coat that render them physically dormant; (ii) dormancy is effectively broken by hot-water treatment (>90 °C) and summer–autumn climatic conditions, both in the laboratory and in the field; (iii) imbibition occurs only at the lens, which is the primary water gap; (iv) germination in the soil is determined by seasonal temperature changes that occur during summer and autumn. Below, we discuss our findings in more detail.

Fully mature, intact seeds of *A. pavonina* did not imbibe water when placed in a wet medium under ambient laboratory conditions. In contrast, seeds that were manually scarified or treated with hot water readily imbibed water. The results therefore support previous findings of Brancalion *et al.* (2010) and Bahar (2015) that seeds of *A. pavonina* have an impermeable seed coat, and thus PY. Dormancy could only be effectively broken when treated with hot water at temperatures exceeding 90 °C. Such high temperatures are not characteristic of natural conditions, although they have been known to occur in the study region during fire (G. K. Jaganathan *et al.*, unpubl.). Conversely, seeds stored at room temperatures or in dry storage did not break dormancy even after 2 years. Likewise, rapid temperature fluctuations did not render seeds permeable to water. These results support similar findings in other PY species, e.g. *Trifolium subterraneum* (Hagon 1971). No untreated mature seeds germinated; however, the germination of hot-water-treated seeds was above 80 % at all incubation temperatures ranging from 15 to 30 °C, except at 20/25 °C (complete darkness) where 79 % of the seeds germinated. These temperature ranges occur during middle or late autumn season and we noted several seeds germinated in the field during this period.

We explored the importance of summer temperatures followed by autumn temperature regimes in breaking dormancy. The results showed that, when *A. pavonina* seeds were only incubated at summer soil temperatures (15/60 °C), germination rates were poor (3 % and 14 % after 1 and 2 months, respectively). On the other hand, seeds subsequently moved to autumn temperatures (15/45 °C) exhibited significantly higher germination rates (Fig. 3). In particular, 46 % of seeds became permeable to water when incubated for 2 months at summer temperatures followed by 1 month at autumn temperatures (Fig. 3). In the natural environment, particularly in the tropics, the requirement of seasonal temperature changes to break dormancy is an important adaptation that prevents seeds from germinating in the seasons that do not favour continued growth of seedlings, particularly when exposed to ‘false cues’, such as isolated summer showers. Germination at these times is likely to result in drought-induced seedling mortality (Baskin and Baskin 2014).

To our knowledge, this study provides the first qualitative description of the anatomical structures that form the primary water gap in a species with mimetic seeds. When *A. pavonina* seeds were exposed to hot water, cells of the palisade layer became dislodged, causing the lens to open (Fig. 4B). Gama-Arachchige *et al.* (2013) suggested the lens acts as a ‘water gap’ in all Mimosoideae species e.g. *A. kempeana* (Hanna 1984), *Leucaena leucocephala* (Serrato-Valenti *et al.* 1995) and *A. lophantha* (Dell 1980). While the terms ‘lens’ and ‘strophiolar plug’ are both used to describe the water-gap structure, we agree with Gama-Arachchige *et al.* (2013) that this structure is best referred to as the ‘lens’, given that it occurs on the opposite side of the micropyle and adjacent to the hilum (Fig. 4; Dell 1980; Hanna 1984; Serrato-Valenti *et al.* 1995). The presence of a palisade layer throughout the seed coat is the likely cause of seed impermeability (Fig. 4A).

The structures through which water enters the seed vary taxonomically (Gama-Arachchige *et al.* 2013). The ‘lens’ or ‘pseudo-lens’ primarily acts as the water gap in many Fabaceae species (Gama-Arachchige *et al.* 2013); however, the hilum might also serve as a secondary water entry point. Gama-Arachchige *et al.* (2013) introduced the terms ‘simple’ and ‘compound water-gap complexes’ to describe water entry through ‘one’ and ‘multiple’ structures, respectively. Water-gap complexes were further categorized into Type-I, Type-II and Type-III according to their shape. Type-I water-gap complexes allow water to enter seeds through narrow linear openings occluded by modified elongated palisade cells. Type-II water-gap complexes have circular or narrow linear openings occluded by lid-like structures formed by the palisade cells. In Type-III water-gap complexes, the openings are either narrow linear or circular, occluded by plug-like structures formed by water-impermeable sclerenchyma cells. All Mimosoideae species investigated to date have simple Type-II water-gap complexes, with no secondary water gaps reported (Gama-Arachchige *et al.* 2013). In accordance with this view, we report a simple Type-II water-gap complex in *A. pavonina* as water enters only through the lens (Fig. 5). In *A. pavonina* seeds, the embryo is located below the lens but the lens modulates the speed of water uptake, taking more than 96 h for seeds to fully imbibe water (Fig. 1). This feature allows seeds to germinate during the wet season, thus avoiding germination in response to isolated summer showers that do not supply enough water for complete imbibition.

Previous studies have suggested that high summer temperatures ‘condition’ seeds before cooler autumn temperatures open the water gap (Cook *et al.* 2008; Jaganathan and Liu 2014; Jaganathan *et al.* 2017). Our results support this notion. *Adenanthera pavonina* seeds buried during summer and exhumed in autumn exhibited higher germination rates than seeds buried during autumn and exhumed during early spring (Fig. 7). The location of seeds in the soil also determines the germination rate of many PY species (Quinlivan 1967; Vázquez-Yanes and Orozco-Segovia 1982; Taylor and Ewing 1988). Seeds of *A. pavonina* buried at 3 cm had significantly higher germination rates compared with seeds buried at 7 cm (Fig. 7; Table 1). The difference in germination rate could partly be attributed to the soil temperatures. Seeds buried at 3 cm experienced higher soil temperatures and greater fluctuations compared with seeds buried at 7 cm (Fig. 7). Many seeds buried at 3 cm depth germinated in the laboratory when exhumed during autumn, having experienced both high summer temperature and nearly 1 month of low autumn temperature fluctuation. Whilst these conditions satisfied dormancy breaking requirements, dormancy broken seeds did not germinate in the field because conditions required for germination were not met. Water is an important requirement for germination to occur in non-dormant seeds and the rainy season at our study site begins in late September with only occasional rainfall during August (Indian Meteorological Department).

Many PY species form long-term soil seed banks with germination spread out over many years (Egley and Chandler 1983; Long *et al.* 2015). The high percentage of *A. pavonina* seeds remaining dormant but viable in the soil suggests this species also forms long-term soil seed banks. Further, our results demonstrate that, even after 2 years in the soil, only 60 % of seeds could germinate. Remaining seeds would presumably require a longer period to break dormancy and germinate. Future studies should explore the germination ecology of other species with mimetic seeds from different climatic regimes.

## Conclusion

In conclusion, we show that PY in *A. pavonina* can be broken by several experimental treatments, including hot water and mechanical scarification. However, temperatures above 90 °C are required to effectively break the dormancy of most seeds. Dormancy break is characterized by the opening of the lens (Type-II simple) due to dislodgement of the palisade layer, through which water first enters the seeds. In natural conditions, dormancy break is regulated by summer and autumn temperature changes. Seeds buried at 3 cm depth had significantly higher germination rates than those buried at 7 cm depth. However, irrespective of burial depth, seeds exhumed after autumn were more likely to be permeable to water, indicating that germination primarily occurs in autumn.

## Sources of Funding

Financial support by Chinese Government (grant number 2016M601620) and National Science Foundation of China (NSFC) (grant number 31750110474) is gratefully acknowledged.

## Conflict of Interest

None declared.

## Contributions by the Authors

G.K.J. designed and performed experiments, analysed data and wrote the manuscript. K.J.Y. assisted in writing and data analysis; M.B. assisted in revising the manuscript.
